# Does ethnicity matter in risk and protective factors for suicide attempts and suicide lethality?

**DOI:** 10.1371/journal.pone.0175752

**Published:** 2017-04-20

**Authors:** Carol C. Choo, Keith M. Harris, Peter K. H. Chew, Roger C. Ho

**Affiliations:** 1 College of Healthcare Sciences, James Cook University, Singapore, Singapore; 2 School of Medicine, University of Tasmania, Hobart, Tasmania, Australia, and School of Psychology, University of Queensland, St Lucia, Queensland, Australia; 3 Yong Loo Lin School of Medicine, National University of Singapore, Singapore, Singapore; Chiba Daigaku, JAPAN

## Abstract

This study explored ethnic differences in risk and protective factors for suicide attempts, for the major ethnic groups in Singapore, and ethnic differences in prediction of lethality. Three years of medical records related to suicide attempters (*N* = 666) who were admitted to the emergency department of a large teaching hospital in Singapore were subjected to analysis. Of the sample, 69.2% were female, 30.8% male; 63.8% Chinese, 15.8% Indian, and 15.0% Malay. Indians were over-represented in this sample, as compared with the ethnic distribution in the general population. Ages ranged from 10 to 85 years old (*M* = 29.7, *SD* = 16.1). Ethnic differences were found in risk and protective factors, and perceived lethality of suicide attempts. All available variables were subjected to regression analyses for Chinese, Indian and Malay attempters to arrive at parsimonious models for prediction of perceived lethality. The findings were discussed in regards to implications in assessment of suicide risk and primary prevention for the multiethnic society in Singapore.

## Introduction

Suicide has become a serious problem worldwide, with an annual global age-standardized suicide rate of 11.4 per 100 000 population [1]. Hospitalizations for attempted suicides occur at a rate of six to seven times that of completed suicides [2]. Attempted suicide is an important predictor of eventual suicide [3], and is a serious public health problem, with significant tolls for psychiatric and healthcare services. Singapore is one of the few countries in the world where suicide attempts are illegal, thus documented police records would give a good gauge of local rate of suicide attempts. According to recent police records in Singapore, there has been increase in suicide attempts to 20.5 per 100,000 population [4].

Lethality of suicide attempts, lethality of methods chosen, and fatality of the outcome are inter-related [5]; and cultural variations have been reported [6–10]. Cultural variations in suicide rates might be explained by underlying ethnic differences for risk and protective factors for suicide attempts. Culture affects the types of stressors, and cultural meaning attributed to precipitants that lead to suicidal behavior, as well as threshold for tolerance of psychological pain [11–14]. The contribution of culture in suicide attempts could be explored by unravelling the negative impact of interacting cultural mechanisms [15]. One example was in the United States, rates for suicidal ideation and attempts in African Americans were lower than Caucasians, which were congruent with the lower rates of completed suicides in African Americans [16]. Protective factors included strong family ties, strong connections to the church, extended kin and social support networks, which promoted social cohesion, shared values, and mutual support. Another example is Singapore, which is a multiethnic society where the main ethnic groups consist of Malays, Chinese, and Indians; suicide data reveal that Malays have consistently the lowest suicide rates in all age groups [17,18]. The low rates in attempted suicide for Malays are similar to their low rates in suicide deaths, as compared to the other races [19]. It is suggested that protective factors might include effective social support systems within the family and the community, which help to buffer against many of life’s stresses [7,20,17]. In Singapore, Indian females have the highest rates in attempted suicide, but not in suicide deaths, suggesting that suicidal behavior may be seen as a reaction to stress, and may not be reflective of a desire to die [21], but could be linked to lack of coping behaviours [22]. Low medical lethality suicide attempts appeared to be unpremeditated overdoses, with ambivalent suicide intent, located in the family home, and were rescued by family members [8]. It is unclear whether some attempts might be perceived by the attempter to be lethal but without medically lethal outcomes due to prompt interventions by potential rescuers. Although lethality is an important clinical variable often linked to fatality of outcomes of the suicide attempt [5], our understanding of lethality is limited by lack of local research examining inter-relationships between medical lethality, whether medical lethality is linked to attempters’ perception of lethality, features of the attempt, and suicide risk and protective factors. Cultural variations were well reported in suicide statistics [17–18], but specific cultural variations underlying suicide risk and protective factors were not well explained. Such information is important in culture specific risk assessment and in our ultimate aim to prevent death through suicides, but most suicide prediction studies lack accuracy in predictive power and therefore has limited clinical utility [23–24].

Inevitably, people are influenced by their society and culture [25]. Studies of suicide of different ethnicities enable us to understand the cultural meaning that various ethnicities possess towards suicide [12], protective factors stemming from both internal and external resources available to help the individuals to cope, and the support that they can expect to receive from the community [26]. A review of relevant literature shows that many risk and protective factors were related to suicide deaths and suicide attempts in both Western and Asian studies, listed in the next two paragraphs. However, there is a lack of large scale research concurrently examining ethnic differences in suicide risk and protective factors, as well as lethality of suicide attempts and related features of the attempts in Asia, which is important for informing targeted assessment and intervention strategies in this region, and to inform targeted primary prevention strategies in the community. Singapore’s multiethnic society offers a unique opportunity to study ethnic influences on suicide risk and protective factors, as well as ethnic differences in lethality of suicide attempts.

The current study aims to explore ethnic influences on risk and protective factors for suicide attempts, as well as lethality of attempts for the major ethnic groups in Singapore. Based on past evidence in both Western and Asian studies, analysis will be conducted on the following available variables, which are salient in literature with good clinical relevance. Risk factors include: living alone [27], unemployment [27–29], financial problem [30], physical illness [31], mental illness [29–30,32], alcohol/ drug use [6,33] interpersonal conflict [28–30], protective factors include: presence of dependents [34], emotional support [35], willingness to seek help [36–37], resolution of precipitants [38], religion [39], regret of the attempt [40], and positive future planning [41]. It is hypothesized that there will be ethnic differences in risk and protective factors for suicide attempts.

This study then aims to refine suicide risk assessment by exploring ethnic differences in suicide lethality, and prediction of lethality of suicide attempts in the major ethnic groups in Singapore. Available variables will be used to predict lethality. Lethality will be measured using two dimensions, namely medical officer’s judgement of medical severity and patients’ perception of the lethality of their attempts. This could give a more comprehensive perception on lethality as attempts perceived as lethal might result in different medical consequences due to various factors outlined below. Based on past evidence in both Asian and Western suicide research, the following variables are hypothesized to be important variables in the prediction of lethality, namely older age [6,20], male gender [42], lower opportunity of rescue [43], effort to hide attempt [44], admission of suicide intent [45], prior planning of attempt [43], as well as risk and protective factors mentioned previously. It is hypothesized that there will be ethnic differences in perceived lethality and medical lethality. Consistent with local suicide data [17–19], compared to Chinese and Indians, fewer Malays attempters will make high lethality attempts [39], and there will be ethnic differences in the prediction of lethality of suicide attempts.

## Method

### Procedure

Ethics approval was obtained from the Domains-Specific Review Board of a large teaching hospital in Singapore and the Human Research Ethics Committee at James Cook University. This study is based on an archival retrospective review of de-identified hospital records of patients who were admitted for a suicide attempt from January 2004 to December 2006. Data was collected from multiple hospital databases related to the suicide attempters who were admitted over the three-year period and this data set is the most comprehensive data set available from the hospital, as such assessment data was not collected prior to and following the stipulated period. Archival data was extracted from the Patient Psychiatric Assessment Form (PPAF). The PPAF includes the Suicide Risk Assessment form, information about the current suicide attempt, as well as information about the patient, and risk and protective factors.

All cases of attempted suicide were assessed by medical officers in the emergency department under the supervision of a consultant psychiatrist, and the interview took approximately 20 minutes. This assessment was part of the protocol standard operating procedure for patients admitted following a medically treated suicide attempt. At the time of the evaluation, the medical officer made a formal psychiatric and/ or medical diagnosis. After the assessment, a management plan was recommended.

The inclusion criterion for the current study were patients who were admitted to the emergency department from January 2004 to December 2006 and were assessed by medical officers using the PPAF. A total of 671 assessment records were examined in the study. From the raw data, cases with more than 5% missing values were deleted. Of the final 666 cases, 69.2% were female, 30.8% were male; 63.8% were Chinese, 15.8% were Indian, 15.0% were Malay and 5.4% were Eurasian. Ages ranged from 10 to 85 years old (*M* = 29.7, *SD* = 16.1). A recent census conducted by the Department of Statistics in Singapore showed 76.8% of the local population were Chinese, 13.9% were Malay, 7.9% were Indian, and 1.4% were other ethnicities (e.g., Eurasian). In comparison to the ethnic distribution in Singapore, the proportion of Indians in the sample of suicide attempters was over-represented. Pearson chi-square test was used to analyze the difference in ethnic proportions in the suicide attempters and in the general population. The proportion of Indians in the sample is about two times greater than its distribution in the population, this difference is significant, χ^2^(3, *N* = 666) = 194.99, *p* < .0001.

In this sample of suicide attempters, 94% did not have a formal diagnosis during the time of evaluation. The majority of victims overdosed in the suicide attempt.

### Measures

#### Suicide risk assessment form

The Suicide Risk Assessment Form (SRAF) is a 2-page questionnaire designed to be conducted as a semi-structured interview by medical officers. The content of the assessment form includes: demographic information such as gender, age, and ethnicity, qualitative details of the current and previous attempts, presence of substances in the bloodstream or urine samples; and psychiatric diagnosis. It documents presence of prior planning, efforts to hide the suicide attempt, admission of suicide intent, presence of last acts, and usage of alcohol with the attempt on dichotomous scales (yes and no). It records the presence of stressors (e.g., work, family, relationships, financial, medical), mental status examination, risk and protective factors, as well as recommended management plan. The risk factors are recorded on discrete dichotomous scales (yes and no) and include: lack of confidantes, living alone, unemployment, financial problem, mental illness or suicide in the family, alcohol or drug abuse, history of mental illness, interpersonal conflict, and poor coping; the protective factors are recorded on discrete dichotomous scales (yes and no) and include: presence of dependents, emotional support, willingness to seek help, resolution of precipitant, religion, regret, and positive future planning. It assesses patient’s perceived lethality of the attempt on a 3-point scale (not lethal, moderately lethal, very lethal), medical officer’s clinical judgment of the medical severity of the attempt on a 4-point scale (high, moderate, low, no actual attempt), and opportunity for rescue on a 3-point scale (high, moderate, low). It measures the medical officer’s clinical evaluation of current suicide risk on a 4-point scale (low, low to moderate, moderate to high, high). This checklist was devised for the collation of information deemed important for clinical usage in suicide risk assessment and recommendation of management plan, and psychometric properties were not available.

## Results

Before the main analyses were conducted, the data was screened and examined for accuracy of data entry, missing values, and fit between its distribution and the assumptions of the analyses. Cases with more than 5% of missing values were deleted, leaving 666 cases for analysis. The remaining cases with missing values were imputed using the Expectation Maximization logarithm through SPSS v. 21 (IBM Corp.).

A series of chi square analyses was conducted to examine the association between ethnicity with risk and protective factors. S1 Table shows significant ethnic differences for one of the ten risk factors and two of the seven protective factors. Of Chinese attempters, 33.4% had mental illness, this proportion was higher in comparison with 16.2% of Indians and 12% of Malays. For protective factors, 60% of Malay attempters reported presence of religious beliefs, this proportion was higher in comparison with 44.8% of Indians and 26.6% of Chinese. Of the Chinese attempters, 37.4% reported resolution of precipitants, this was lower in comparison with the proportion in the Malays (50%) and Indians (62.9%). The effect sizes associated with the relationships, as measured by Cramer’s V, ranged from .20 to .27, which suggested a small to moderate effect.

The significant factors from S1 Table were subjected to further analysis by comparing the results of the current study (of suicide attempters) to available results from population studies (of non-suicide attempters) [46–47]. Two chi square analyses was conducted to examine the association between ethnicity and suicide/non-suicide attempters in regards to religious belief and history of mental illness. The results are presented in S2 Table. The results indicated significant associations between ethnicity and suicide/non-suicide attempters. Specifically, there was a higher percentage of Chinese suicide attempters with mental illness. There was also a lower percentage of suicide attempters across all races with a religious belief.

Chi square analysis was used to test the hypothesis that there are ethnic differences in perceived and medical lethality. S3 Table shows a significant difference in perceived lethality among the major ethnic groups. Compared to the Chinese and Indians, less Malays made attempts with high perceived lethality. The ethnic groups did not differ significantly on medical lethality. The ethnic groups were thus separately analyzed for perceived lethality.

To examine the prediction of perceived lethality, firstly, correlation analysis was used to examine the interrelationships between the variables. The dependent variable was perceived lethality. The independent variables included all available predictors. To arrive at a parsimonious model for the prediction of lethality, the variables that were significantly correlated with perceived lethality at *p* < .001 were eligible for entry into a regression analysis model together with the demographic factors of age and gender (these demographic factors were well established in studies on both Asian and Western samples [48–49]. Due to the large quantity of available variables, backward-stepwise procedure was used to condense the variables. Variables were eliminated from the model based on likelihood ratio tests. Test assumptions were carefully checked and any violations noted where appropriate. The dependent variable was recoded into the dichotomous variable of 0 = low lethality and 1 = moderate and high lethality attempts. Regression models were examined to look at the likelihood that Chinese, Indians and Malays would make high perceived lethality attempts. Results are shown in S4 Table. For the Chinese, the final model was statistically significant, χ^2^(2, *N* = 419) = 164.76, *p* < .0001, indicating that the model was able to distinguish between attempts with high and low perceived lethality. The final model explained between 36.9% (Cox and Snell *R*^2^) and 51.0% (Nagelkerke *R*^2^) of the variance in perceived lethality, and correctly classified 84.1% of the cases; 86.4% of low lethality and 79.5% of high lethality attempts were correctly predicted. As shown in S4 Table, high perceived lethality for Chinese suicide attempters was predicted by admission of suicide intent and low opportunity for rescue. The odds ratio for admission of suicide intent was 25.83, indicating that those who reported suicide intent were about 26 times more likely to make high lethality attempts compared to those who did not report suicide intent, controlling for other factors in the model. The odds ratio for rescue was .41, indicating that those who had opportunity for rescue were .41 times less likely to make attempts with high perceived lethality. For Malays, the final model for the prediction of high perceived lethality attempts was statistically significant, χ^2^(1, *N* = 95) = 14.67, *p* < .0001, indicating that the model was able to distinguish between attempts with high and low perceived lethality. The final model explained between 15.5% (Cox and Snell *R*^2^) and 24.3% (Nagelkerke *R*^2^) of the variance in perceived lethality, and correctly classified 79.3% of the cases; 81.2% of low lethality and 69.2% of high lethality attempts were correctly predicted. S4 Table shows that high perceived lethality in Malay suicide attempters was predicted by admission of suicide intent. The odds ratio was 8.62, indicating that Malays who reported suicide intent were about 9 times more likely to make attempts with high perceived lethality than those who did not report suicide intent, controlling for other factors in the model. For Indians, the final model for the prediction of high perceived lethality attempts was statistically significant, χ^2^(1, *N* = 104) = 25.03, *p* < .0001, indicating that the model was able to distinguish between attempts with high and low perceived lethality. The final model explained between 24.3% (Cox and Snell *R*^2^) and 32.9% (Nagelkerke *R*^2^) of the variance in perceived lethality, and correctly classified 77.9% of the cases; 87.3% of low lethality and 62.9% of high lethality attempts were correctly predicted. As shown in S4 Table, the odds ratio for intent was 11.60, indicating that Indians who reported suicide intent were about 12 times more likely to make attempts with high perceived lethality than those who did not report suicide intent, controlling for other factors in the model.

## Discussion

This study aimed to explore ethnic differences on risk and protective factors for suicide attempts in the major ethnic groups in Singapore, ethnic differences in lethality of suicide attempts and prediction of lethality in the major ethnic groups. Three years of medical records of 666 suicide attempters were analyzed. As hypothesized, ethnic differences were seen in suicide risk and protective factors. Findings from the current study uncover the cultural strengths and vulnerabilities underlying the ethnic distributions for suicide statistics in Singapore. In the current study, more Chinese attempters reported a history of mental illness and less Chinese attempters reported resolution of precipitants, positive planning, religious belief, and regret of the attempt, compared to the Indians and Malays. Significant factors of history of mental illness and religious belief were compared with available data from national population studies. In particular, there was a higher percentage of Chinese suicide attempters with history of mental illness and a lower percentage of suicide attempters across all races with a religious belief. This pattern of comparatively heightened risk and diminished protection is congruent with the preponderance of Chinese suicides in Singapore. The results from the current study are consistent with Chia’s [26], who reported that a risk factor for young Chinese is mental illness, and congruent with the prevalence of a prior mental illness for suicide deaths in Singapore [50]. They also do not have religious beliefs with strong sanctions against suicide, consistent with a review of recent literature showing that religion is a common purveyor of cultural sanctions regarding the acceptability of suicides [51–52].

The finding that more Malays reported religious belief and presence of dependents as possible protective factors is consistent with previous literature which highlighted that the Malay community in Singapore is more family oriented and their religious beliefs are protective [26,53–54].

The hypothesis for ethnic differences in lethality was partially supported. As hypothesized, ethnic patterns were seen in lethality. Compared to Indians and Chinese, less Malays made attempts with high perceived lethality. This is congruent with suicide statistics in Singapore; Malays have consistently the lowest rates in suicide deaths and suicide attempts [18,55]. Although the ethnic difference in perceived lethality was significant, the ethnic difference in medical lethality was not significant. Chinese made up the largest proportion while Indians made up the lowest proportion of high medical lethality attempts, but this difference was not significant. In-depth qualitative interviews might be helpful to uncover the intricate patterns and further research could be conducted to examine possible interactions between gender and ethnicity in suicide lethality. Further research is needed to further investigate if it might be possible that the observation could support Mehta’s [8] finding that many Indian females made suicide attempts in the midst of interpersonal conflicts at times of emotional turmoil, which could have precipitated them to make attempts with heightened perceived lethality, but their attempts were often impulsive, and unplanned, and the attempt usually happened at home with family members present, so opportunity for rescue was high, and with access to medical interventions, medical lethality was low.

There are ethnic differences in the regression models. For Chinese, high perceived lethality was predicted by admission of intent and low opportunity for rescue. For Indians and Malays, admission of suicide intent was the only significant predictor of perceived lethality. The current study has highlighted the importance of comprehensive suicide assessment which includes a clinical interview that uncovers the suicide intent of the client, as to whether the client’s intention was to die, across the ethnic communities.

The findings that there are ethnic differences in suicide risk and protective factors have implications for suicide interventions and primary prevention. Interventions could be more targeted, with greater service level collaborations to reach out to vulnerable communities. Suicide prevention efforts could be targeted at raising resilience to buffer against the identified risk factors and strengthen the identified vulnerabilities. Psycho-education, screening and interventions for mental health promotion could be encouraged in the Chinese community. Counselling and crisis interventions could be more targeted towards resolution of precipitants and positive future planning, especially for Chinese clients. For both Chinese and Indian communities, campaigns could be held to highlight the importance of family, strengthen family resilience, and to promote spiritual / religious values that will discourage suicide. Such strategies focusing on family functioning and connectedness, and problem solving skills are consistent with interventions suggested by previous literature for suicide prevention in Asian youths [56].

Limitations to the study include the following: Assessments are in English as English is the official language in Singapore. It is possible that language barrier may impact data collection and future studies could look at translation into other languages e.g. Mandarin. There are no available records for immigration status available for analysis in the current study. Future studies could incorporate this information, in view of reviews of recent research highlighting contribution of acculturation stress to suicide risk [12,49,57]. A lack of a control group also limits how the data could be interpreted. Future research could compare data between suicide attempters and matched controls in the normal population for better interpretation of data.

Other limitations of the study include the reliance on self-report and the brief nature of the assessment, as well as the usage of single items on dichotomous scales, which place constraints on the depth of the information obtained. Future research could employ qualitative interviews to explore the relationships and processes underpinning risk and protective factors, suicide intent, opportunity for rescue, and other factors that might be relevant for understanding lethality of suicide attempts in different ethnic communities.

In conclusion, the findings have implications for informing our efforts in suicide assessment and primary prevention in the multi-ethnic society in Singapore. Using ethnic-specific assessment and interventions that are substantiated by empirical findings from current research in the local population, clinicians may take a step forward in utilizing the scientist-practitioner model in their evidence based practice [58]. A challenge common to cultural diversity research is the balance between cultural specificity and generalizability [12], the current study not only highlighted the importance of the assessment of suicide intent in suicide risk assessment across the ethnic communities, but also suggests specific interventions that could be tailored for the ethnic communities. This study adds to the current literature on culture and suicide research, and draws further focus to the importance of training culturally competent clinicians who are sensitive to culture influences on suicide risk.

## Supporting information

S1 Table Ethnic differences in known risk and protective factors. (DOCX)

S2 Table Ethnic differences in suicide attempters and general population for mental illness and religious belief. (DOCX)

S3 Table Ethnic difference in suicide attempts with high medical and perceived lethality. (DOCX)

S4 Table Logistic regression predicting likelihood of suicide attempts with high perceived lethality in the major ethnic groups. (DOCX)
