# Supplementary material for: Does ethnicity matter in risk and protective factors for suicide attempts and suicide lethality?
Source: PLoS One. 2017 Apr 20;12(4):e0175752. doi: 10.1371/journal.pone.0175752 (PMC5398550; doi:10.1371/journal.pone.0175752)
Supplement: S1 Table — (DOCX) [file pone.0175752.s001.docx]

S1 Table. Ethnic Differences in Known Risk and Protective factors.

|  | Factors | Chinese | Indian | Malay | χ ^2^ | Cramer’s V |
| --- | --- | --- | --- | --- | --- | --- |
| Risk factors |  |  |  |  |  |  |
|  | History of mental illness | 33.4% | 16.2% | 12.0% | 26.41* | .21* |
|  | Living alone | 11.8% | 15.2% | 8.0% | 2.59 | .06 |
|  | Familial mental illness/suicide | 12.5% | 8.6% | 8.0% | 2.46 | .06 |
|  | Alcohol/ drug abuse | 21.2% | 15.2% | 18.0% | 2.09 | .06 |
|  | Interpersonal conflicts | 48.5% | 47.6% | 56.0% | 2.02 | .06 |
|  | Lack of confidantes | 36.5% | 31.4% | 32.0% | 1.39 | .05 |
|  | Serious physical illness | 73.7% | 5.7% | 4.0% | 0.98 | .04 |
|  | Habitual poor coping | 39.5% | 35.2% | 36.0% | 0.91 | .04 |
|  | Serious financial problem | 70.2% | 16.3% | 14.0% | 0.60 | .03 |
|  | Unemployment | 18.6% | 19.0% | 19.0% | 0.02 | .01 |
| Protective factors |  |  |  |  |  |  |
|  | Religious belief | 26.6% | 44.8% | 60.0% | 45.14* | .27* |
|  | Resolution of precipitants | 37.4% | 62.9% | 50.0% | 24.11* | .20* |
|  | Has dependents | 56.5% | 59.0% | 76.0% | 12.91 | .14 |
|  | Expressed regret | 72.2% | 83.8% | 83.0% | 9.46 | .12 |
|  | Positive planning | 70.8% | 80.0% | 80.0% | 6.02 | .10 |
|  | Willing to seek help | 68.2% | 74.3% | 69.0% | 1.46 | .05 |
|  | Emotionally supported | 74.4% | 75.2% | 80.0% | 1.39 | .05 |

*Note.* *N* = 666*, df* = 2. Percentages are percentages of the group endorsing that factor, e.g., 33.4% of Chinese, 16.2% of Indian and 12% of Malay suicide attempters had history of mental illness. Due to the number of analyses conducted, the alpha level was controlled for using the Bonferroni adjustment (i.e., adjusted alpha = .05/17 analyses = .003).

**p* < .003.
