# Supplementary material for: Does ethnicity matter in risk and protective factors for suicide attempts and suicide lethality?
Source: PLoS One. 2017 Apr 20;12(4):e0175752. doi: 10.1371/journal.pone.0175752 (PMC5398550; doi:10.1371/journal.pone.0175752)
Supplement: S2 Table — (DOCX) [file pone.0175752.s002.docx]

S2 Table. Ethnic Differences in Suicide Attempters and General Population For Mental Illness and Religious Belief.

|  | Factors | Chinese | Indian | Malay | χ ^2^ | Cramer’s V |
| --- | --- | --- | --- | --- | --- | --- |
| Mental illness |  |  |  |  |  |  |
|  | Suicide attempters | 33.4% | 16.2% | 12.0% | 151.27* | .30* |
|  | Non-suicide attempters^a^ | 17.4% | 17.8% | 15.1% |  |  |
| Religious belief |  |  |  |  |  |  |
|  | Suicide attempters | 26.6% | 44.8% | 60.0% | 47.64* | .00* |
|  | Non-suicide attempters^b^ | 78.2% | 98.9% | 99.8% |  |  |

*Note.* *df* = 2. Percentages are percentages of the group endorsing that factor, e.g., 33.4% of Chinese, 16.2% of Indian and 12% of Malay suicide attempters had history of mental illness. Due to the number of analyses conducted, the alpha level was controlled for using the Bonferroni adjustment (i.e., adjusted alpha = .05/2 analyses = .025).

^a^Results from Fones et al. (1998) [46].

^b^Results from Singapore Department of Statistics (2010) [47].

**p* < .025.
