# Supplementary material for: Does ethnicity matter in risk and protective factors for suicide attempts and suicide lethality?
Source: PLoS One. 2017 Apr 20;12(4):e0175752. doi: 10.1371/journal.pone.0175752 (PMC5398550; doi:10.1371/journal.pone.0175752)
Supplement: S3 Table — (DOCX) [file pone.0175752.s003.docx]

S3 Table*.* Ethnic Difference in Suicide Attempts with High Medical and Perceived Lethality.

| Variable | Chinese | Indian | Malay | χ ^2^ | Cramer’s V |
| --- | --- | --- | --- | --- | --- |
| Perceived lethality | 43.8% | 47.6% | 30.0% | 8.92* | .12 |
| Medical Lethality | 52.7% | 42.9% | 48.0% | 3.52 | .07 |

*Note.* *N* = 666. *df* = 3

**p = .*03*.*
