# Supplementary material for: Does ethnicity matter in risk and protective factors for suicide attempts and suicide lethality?
Source: PLoS One. 2017 Apr 20;12(4):e0175752. doi: 10.1371/journal.pone.0175752 (PMC5398550; doi:10.1371/journal.pone.0175752)
Supplement: S4 Table — (DOCX) [file pone.0175752.s004.docx]

S4 Table. Logistic Regression Predicting Likelihood of Suicide Attempts with High Perceived Lethality in the Major Ethnic Groups.

| Predictor | |  | |  | |  | |  | | 95% CI for *OR* | | | |  |
| --- | --- | --- | --- | --- | --- | --- | --- | --- | --- | --- | --- | --- | --- | --- |
| Variable | | *B* | | *SEB* | | Wald | | *OR* | | *LL* | | *UL* | |  |
| Chinese^a^ |  | |  | |  | |  | |  | |  | |  | |
|  | Intent | | 3.25 | | .30 | | 115.22**** | | 25.83 | | 14.26 | | 46.77 | |
|  | Rescue | | -0.89 | | .32 | | 7.59* | | 0.41 | | 0.22 | | 0.77 | |
|  | Constant | | -0.55 | | .58 | | .00 | | 0.58 | |  | |  | |
| Malays^b^ |  | |  | |  | |  | |  | |  | |  | |
|  | Intent | | 2.51 | | .59 | | 13.45**** | | 8.62 | | 2.73 | | 27.23 | |
|  | Constant | | -2.23 | | .13 | | 27.04 | | 0.12 | |  | |  | |
| Indians^c^ |  | |  | |  | |  | |  | |  | |  | |
|  | Intent | | 2.45 | | .54 | | 21.01**** | | 11.60 | | 4.07 | | 33.11 | |
|  | Constant | | -1.31 | | .31 | | 17.46**** | | 0.27 | |  | |  | |

*Note.* ^a^*N* = 419. ^b^*N*= 95. *N*^c^ = 104. CI = confidence interval, *OR* = odds ratio, *LL* = lower limit, *UL* = upper limit, Rescue = opportunity for rescue, Intent = admission of suicide intent.

**p* < .05. *****p* < .0001.
